# Experimental Analysis of the Influence of Drill Point Angle and Wear on the Drilling of Woven CFRPs

**DOI:** 10.3390/ma7064258

**Published:** 2014-05-30

**Authors:** Norberto Feito, José Díaz-Álvarez, Antonio Díaz-Álvarez, José Luis Cantero, María Henar Miguélez

**Affiliations:** 1 Department of Mechanical Engineering, Universidad Carlos III de Madrid, Avda. Universidad 30, Leganés, Madrid 28911, Spain; E-Mails: nfeito@db.uc3m.es (N.F.); andiaza@ing.uc3m.es (A.D.-Á.); jcantero@ing.uc3m.es (J.L.C.); 2 Department of Bioengineering and Aerospace Engineering, Universidad Carlos III de Madrid, Avda. Universidad 30, Leganés, Madrid 28911, Spain; E-Mail: jodiaz@ing.uc3m.es

**Keywords:** drilling, woven CFRPs, surface quality, wear

## Abstract

This paper focuses on the effect of the drill geometry on the drilling of woven Carbon Fiber Reinforced Polymer composite (CFRPs). Although different geometrical effects can be considered in drilling CFRPs, the present work focuses on the influence of point angle and wear because they are the important factors influencing hole quality and machining forces. Surface quality was evaluated in terms of delamination and superficial defects. Three different point angles were tested representative of the geometries commonly used in the industry. Two wear modes were considered, being representative of the wear patterns commonly observed when drilling CFRPs: flank wear and honed cutting edge. It was found that the crossed influence of the point angle and wear were significant to the thrust force. Delamination at the hole entry and exit showed opposite trends with the change of geometry. Also, cutting parameters were checked showing the feed’s dominant influence on surface damage.

## 1. Introduction

Carbon Fiber Reinforced Polymer (CFRP) composites combine fatigue and corrosion resistance, light weight and high specific stiffness and strength. These properties make CFRPs suitable for a wide range of structural applications [1]. Within this family of materials, woven graphite fiber epoxy composites have been extensively used in aerospace, automotive and civil applications. Woven CFRPs exhibit higher strength-to weight ratio and higher fracture toughness than unidirectional composites [2].

Although composite components are manufactured close to the final shape, they usually require machining operations in order to achieve dimensional tolerances and assembly specifications. In most cases the component is drilled previously to mechanical joining [3]. Drilling operations of woven carbon composite CFRPs should be designed to be productive processes ensuring the quality of the resultant component. This operation is performed in a high value component, the susceptibility of the composite to undergo machining-induced damage highlights the importance of controlling the process. The composite is exposed to the generation of damage during processing, mainly delamination. This phenomenon is related to machining parameters and drill geometry. Drilling operations of CFRPs involve strong tool wear due to the presence of hard fibers; wear progression leads to variations of the initial geometry of the drill [4].

Various authors have studied CFRPs drilling, mainly in the case of unidirectional composites. A brief summary of the contributions focusing on woven CFRP drilling is presented in the following paragraphs.

Karpat* et al.* [5] analyzed the drilling performance of the double point angle drill for woven CFRP laminates. Uncoated carbide drill and diamond coated carbide drills with different drill point angles were tested in drilling experiments of thick fabric woven CFRP laminates. The feed was more influential than cutting speed on damage generation. At elevated feed rates, the hole diameter tolerance was observed to be more critical than the hole exit delamination.

The influence of the drill point angle on the machining forces and the drill hole quality (in terms of delamination, fraying and burr formation) was analyzed in [6] for woven CFRPs. Increased point angles resulted in enhanced thrust force; however, the torque remains almost constant. The quality at the hole entrance was enhanced when increasing point angles while it was poorer at the exit. The increment of cutting speed lead to negligible differences in the hole quality but resulted in increased thrust forces and decreased drilling torques. This behavior was also observed when drilling cross-ply composite materials with twist drill bits [7]: point angle equal to 120° produced less delamination at hole entry than point angle equal to 85° even though the thrust force was higher in the former case.

The influence of different types of tool geometry when drilling unidirectional CFRP [0/90]_13_ was analyzed in [8]. “SPUR” drill bit (commonly used for wood materials) gave the best results causing small damage extension in the hole perimeter. On the other hand, a twist drill presented higher delamination located at the hole entrance. Similar tests were carried out on unidirectional CFRPs in [9] showing no advantage for step drill when compared with commercial drill bit, which reduced the surface damage.

On the other hand, wear evolution influences drill geometry. The initial design of the fresh tool is modified because of wear progression and, consequently, the effective cutting geometry is varied.

Mayuet* et al.* [10] carried out drilling tests of woven CFRP with conventional carbide drill geometry. It was demonstrated that the abrasion due to the presence of hard fibers was the dominant wear mechanism. The matrix adhesion had a much lower effect. The influence of wear in hole quality was demonstrated since delamination at the hole exit increased significantly as the number of machined holes increased.

Illiescu* et al.* [11] analyzed the influence of feed rate and tool wear in drilling of woven CFRPs with coated and uncoated drills. The feed rate and tool wear were the most significant factors affecting the thrust force. The torque was much less sensitive to wear than the thrust force. The contact length and the axil force applied at the cutting edge were the main factors involved in wear evolution, in both cases considered (coated and uncoated drills).

Shyha* et al.* [12] analyzed the drilling of small holes (1.5 mm); with two different geometries: conventional twisted and stepped. Delamination at the hole entrance was higher for the stepped drill than for the twisted drill. Increases in the feed rate also lead to delamination enhancement. However, the increment of point angle, from 118° to 140°, lead to decrease of delamination.

Rawat and Attia [13] analyzed the tool wear mechanisms of carbide tools in high speed drilling (10,000–15,000 rpm) of woven CFRPs. Fracture (chipping) at the beginning of drilling process and subsequent abrasion were the main wear mechanisms. Abrasive wear on the flank face of the primary cutting edge was stronger than the wear at the rake face.

The influence of cutting edge rounding (CER) and its correlation with surface damage in drilling of woven CFRPs was analyzed in [14]. A correlation between delamination, machining forces and cutting edge rounding was found. Numerical analysis of the influence of CER in the elemental case of orthogonal cutting has been developed by the authors in [15]. The interest of analyzing drilling-induced delamination has motivated the recent development of complex models for drilling. These numerical models have shown good correlation between measured and predicted torque and thrust force as well as delamination extension [16,17].

Although drilling operations of woven composite have motivated the development of different studies, it is still a challenge to advance the comprehension of the effect of tool geometry and the level of wear. In fact the analysis of the crossed effect of the tool geometry and the geometrical changes due to wear progression has been poorly developed in the literature.

The objective of this paper is analyzing the effect of the drill point angle combined with the geometrical effect of drill wear evolution. Two different types of wear modes were studied: flank wear (commonly identified in the literature as the dominant wear mode) and cutting edge honing (resulting from the transition from new acute to used cutting edge). The effect of cutting parameters (cutting speed and feed rate) were also studied. Resultant thrust force and torque have been evaluated together with surface integrity, analyzed in terms of delamination at the entrance and exit of the hole.

## 2. Experimental Work

## 2.1. Workpiece Material

The material studied in this work is a woven CFRP composite, based on AS-4 carbon fiber and epoxy matrix (55.29% resin content) manufactured by Hexcel Composites (Madrid, Spain). The specimens were cut in plates of 120 mm × 29 mm and 2.2 mm thick, composed of 10 plies with the same fiber orientation. The characteristics and mechanical properties of the workpiece provided by the composite manufacturer are presented in the Table 1, where ρ is density; *E_i_* elastic modulus in the direction *i*; *υ_ij_* Poisson coefficient; *G_ij_* elastic modulus in shear directions; *X_t_*, *Y_t_* and *S_t_* maximum tensile stress in longitudinal and shear directions respectively; *X_c_* and *Y_c_* maximum compressive stress in longitudinal directions.

**Table 1:** Characteristics and mechanical properties of the composite.

ρ	*E*_1_ =* E*_2_	*E*_3_	*υ*_12_	*υ*_13_ =* υ*_23_
1570 Kg/m^3^	68 GPa	10 GPa	0.22	0.49
*G*_12_	*G*_23_ = *G*_13_	*X_t_* =* Y_t_*	*X_c_* =* Y_c_*	*S_t_*
5 GPa	4.5 GPa	795 MPa	860 MPa	98 MPa

## 2.2. Drills

**Figure 1:**
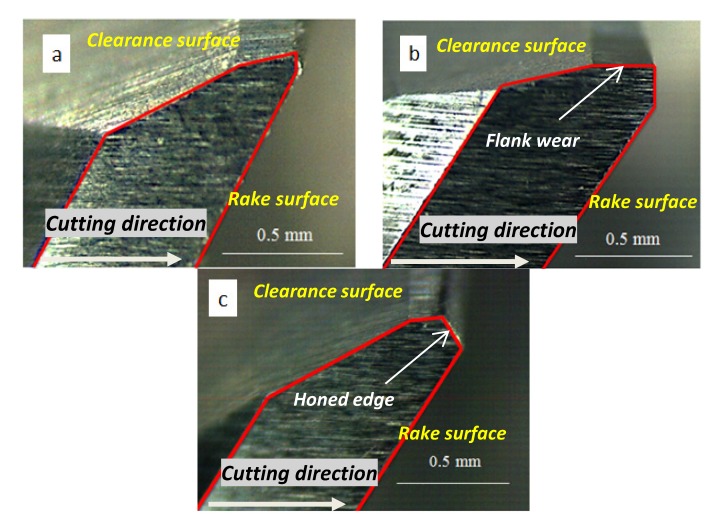
Edge geometry of the drills: (**a**) fresh tool; (**b**) flank wear; and (**c**) honed edge.

Uncoated helicoidal carbide drills recommended by the manufacturer GUHRING for CFRPs drilling were used. Nominal diameter was equal to 6 mm with 30° helix angle. Three different values of the point angle 90°, 118° and 140° were used. Three different stages concerning wear evolution were tested: fresh drill, flank wear equal to 0.3 mm and honed cutting edge with length equal to 0.05 mm (see Figure 1).

The worn geometries were artificially generated using grinding. The level of wear has been stated according to values corresponding to the end of tool life in the literature. Faraz* et al.* [14] reported a value of flank around 0.25 mm corresponding with advanced wear progression. On the other hand, honed edge is an approximation to chipping wear observed in drilling CFRP. As drilling starts chipping appears because acute cutting edges are not able to tolerate high stress. This phenomenon was reported by Rawat* et al.* [13] with an extension of honed edge zone similar to that considered in the present work.

## 2.3. Machining Tests

**Figure 2:**
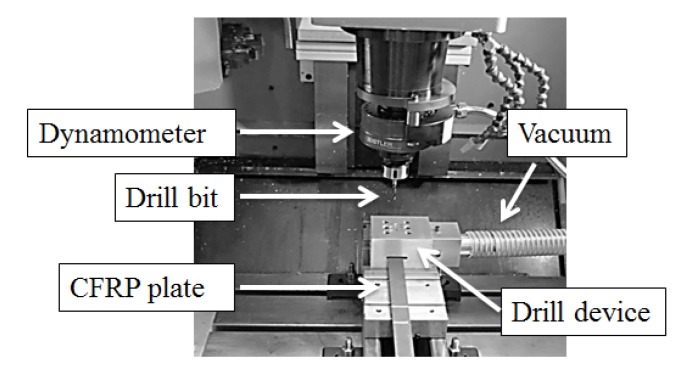
Experimental device for drilling tests.

The drilling tests were carried out on a machining center (B500 KONDIA, Kondia, Elgoibar, Spain). The machining center was equipped with a rotating dynamometer (Kistler 9123C, Winterthur, Switzerland) used for the measurement of the three force components *F_x_*, *F_y_*, *F_z_* and the drive moment *M_z_* on the rotating tool. The acquisition system coupled to the machine tool is shown in Figure 2.

**Figure 3:**
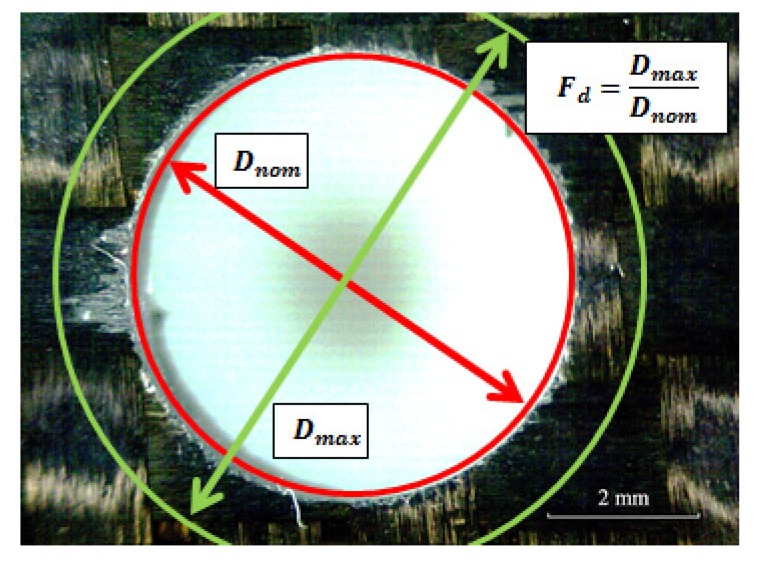
Example of delaminated specimen for calculation of delamination factor *F_d_*.

Drilling induced damage was quantified in terms of the delamination factor (*F_d_*) being the ratio between the maximum diameter of delaminated area and the nominal diameter of the hole (see Figure 3). Other defects induced during machining were analyzed from observation of images of the machined hole obtained with a stereo microscope (Optika SZR, Ponteranica, Italy).

Concerning the cutting conditions, the drilling experiments were conducted without coolant and the cutting parameters summarized in Table 2. Machining operations in composites are commonly carried out in dry conditions because it is required to avoid the composite contamination with the cutting fluid. The machining parameters (cutting speed 25–100 m/min and feed 0.05–0.2 mm/rev) were selected following the recommendations of the drills manufacturer GUHRING for drilling CFRPs.

**Table 2:** Cutting parameters used in drilling tests: *f*, feed and *V*, cutting speed.

Parameter	Range		
*f* [mm/rev]	0.05	0.1	0.15
*V* [m/min]	25	50	100

Drilling tests were performed using a supporting back plate previously drilled with hole diameter equal to 6.2 mm. The use of back plate is commonly desired in industry when drilling plates of composite in order to diminish delamination. The influence of the position of the drilled hole related to the textile float was not considered in this paper. Although this parameter may influence the delamination, the drilling tests were carried out in random relative positions of the drill to the textile float. Observed delamination did not show significant differences for the tests carried out in the same cutting conditions despite the variations of the relative position drilled hole/float, indicating low influence of this parameter.

## 3. Results and Discussion

## 3.1. Thrust Force and Torque

**Figure 4:**
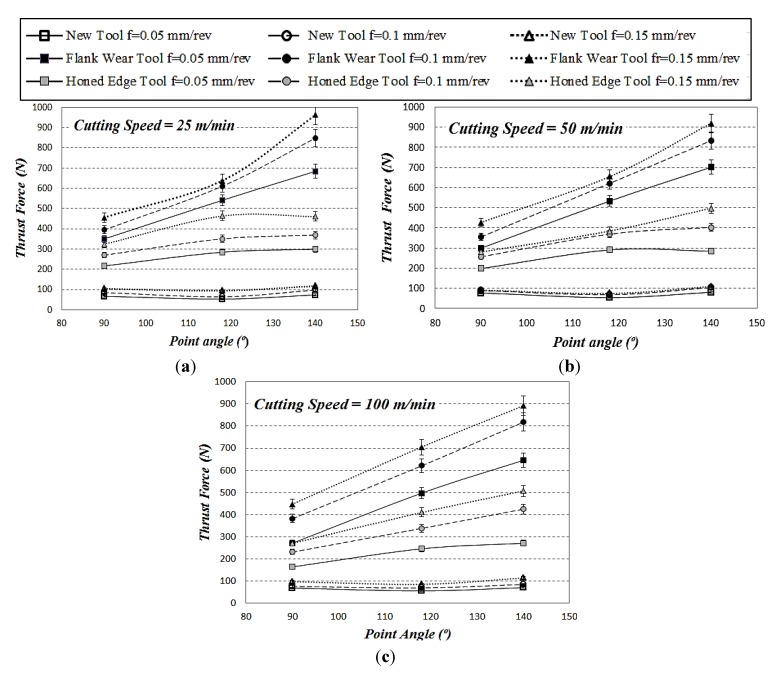
Thrust force for three point angles at different cutting speeds (measurement variations are indicated including error bars): (**a**) cutting speed 25 m/min; (**b**) cutting speed 50 m/min; and (**c**) cutting speed 100 m/min.

The evolution of thrust force and torque with cutting time was recorded using the dynamometer. The maximum level of thrust force obtained during drilling tests is presented in Figure 4. The maximum thrust force obtained with new and worn drills is presented for the different values of point angle and cutting speed considered. It is possible to observe the negligible influence of the point angle on thrust force when fresh drill is used since the projection of the resultant force in the axial direction is the same for all point angles. The increment of feed lead to thrust force enhancement (the maximum values of thrust force obtained with new tools ranged between 50 and 150 N for all cases tested).

Honed tools showed increasing thrust force (around 50%–65%) with the increment of point angle. The thrust force also increases with the point angle when the flank wear geometry is tested. Relative variations around 100% are observed when the angle ranged from 90° to 140°. This trend is observed for all values of cutting speed analyzed.

The enhancement of the feed also resulted in increased thrust force in all cases analyzed. The variation is small for the fresh geometry. However, the increment of thrust force with feed reached increments around 50% and 70% when drilling with flank and honing wear respectively.

The increased values of thrust force due to the use of worn tools are related to enhanced risk of delamination. This trend is observed in the range of cutting speed tested (25–100 m/min). The progression of wear in both cases analyzed (flank wear and honed edge), increases the effect of the feed and the point angle on the thrust force.

In the case of new tool, torque decreased when the point angle increased from 90° to 118° and increased slightly when the angle changed from 118° to 140°. However, for both worn tools, flank wear and honed edge, maximum values of the torque were observed for the point angle 118°. The influence of feed in torque was much higher than in the thrust force, for all cases. The increment of feed lead to torque enhancement in the range 53%–100%.

Concerning the influence of cutting speed, slight decrease of thrust force and torque were observed when the velocity was increased. This trend has been also reported in other works in the literature for woven [12,14] and unidirectional composites [7].

ANOVA analysis presented in Table 3 showed that the main contributing factors for thrust force were tool geometry, point angle and feed rate. For all cases, *p*-value < 0.05 and Test F >> Fα = 5%. The cutting velocity factor did not present a statistical significance because *p*-value > 0.05 and Test F < Fα = 5%. This analysis corroborates the previous discussion concerning the prevalent influence of the worn geometry and the thrust force. On the other hand Davim* et al.* [18] showed the same effect with ANOVA analysis in previous research.

**Table 3:** ANOVA analysis developed for thrust force.

Factors	SS	DF	MS	F	F *	P *
A: Geometry	3.57 × 10^6^	2	1.79 × 10^6^	242.83	3.12	**0.0000**
B: Point Angle	525385	2	262692	35.71	3.12	**0.0000**
C: Cutting Speed	3023.19	2	1511.59	0.21	3.12	0.8147
D: Feed rate	192734	2	96367.1	13.1	3.12	**0.0000**
Residual	529651	72	7356.26	–	–	–
Total	4.82E+06	80	–	–	–	–

SS: Sum of squares; DF: Degrees of freedom; MS: Mean square; F: F-test value; P *: Probability; * Significant at the 5% level.

## 3.2. Surface Quality

The hole quality was evaluated in terms of hole diameter, delamination factor at the entrance and exit of the hole and other qualitative defects related to surface damage. 

The diameter was measured after each drilling test using a micrometer Mitotuyo model 368-101 (Kawasaki, Japan). The values of diameter measured showed a reasonable quality for a drilling operation with a nominal drill diameter equal to 6 mm.

**Figure 5:**
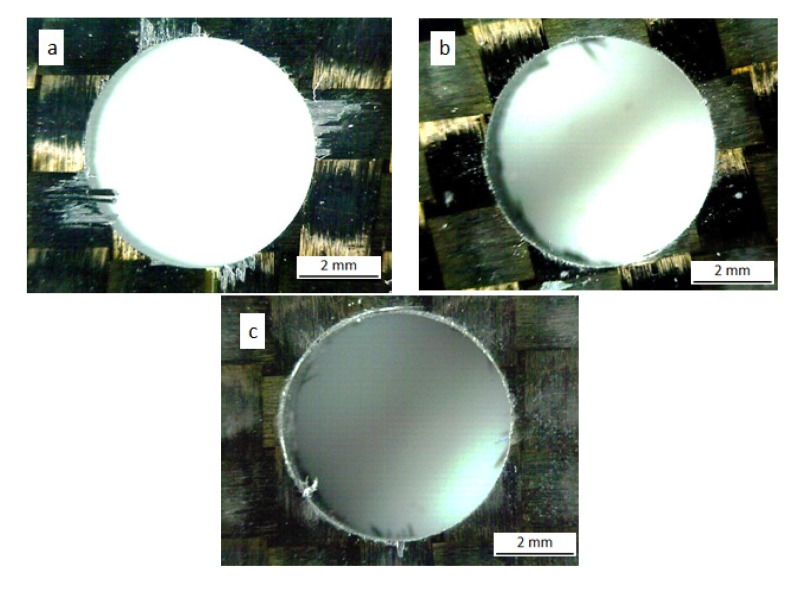
Peel up delamination at the hole entrance for new tool (**a**), flank wear tool (**b**) and honed edge tool (**c**) with point angle 118°, *V* = 50 m/min and *f* = 0.05 mm/rev.

**Figure 6:**
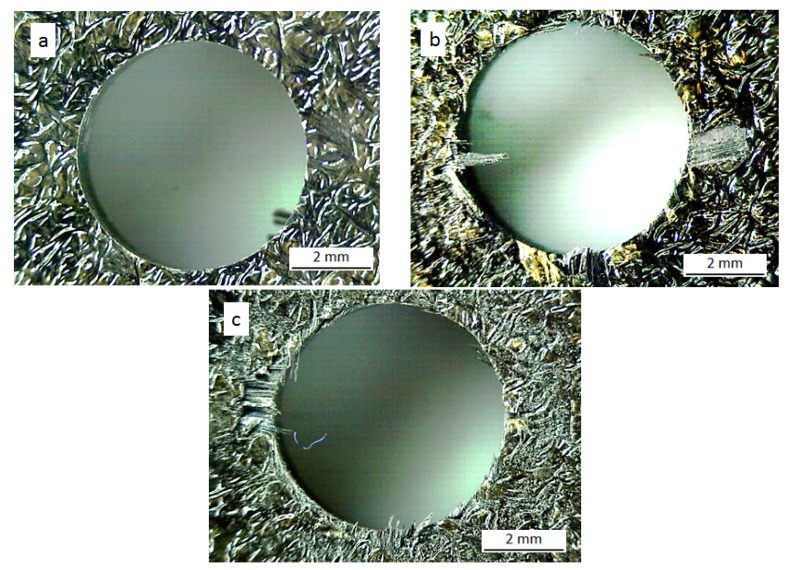
Push out delamination at the hole exit for new tool (**a**), flank wear tool (**b**) and honed edge tool (**c**) with point angle 118°, *V* = 50 m/min and *f* = 0.05 mm/rev.

The delamination factor *F_d_*, was calculated as the ratio between maximum diameter of delaminated area and the nominal diameter of the drill (6 mm). The drilled holes were examined with an optical microscope in order to measure the delaminated area. Examples of delamination at the hole entrance (peel up) and at the exit (push out) are shown in Figure 5 and Figure 6.

**Figure 7:**
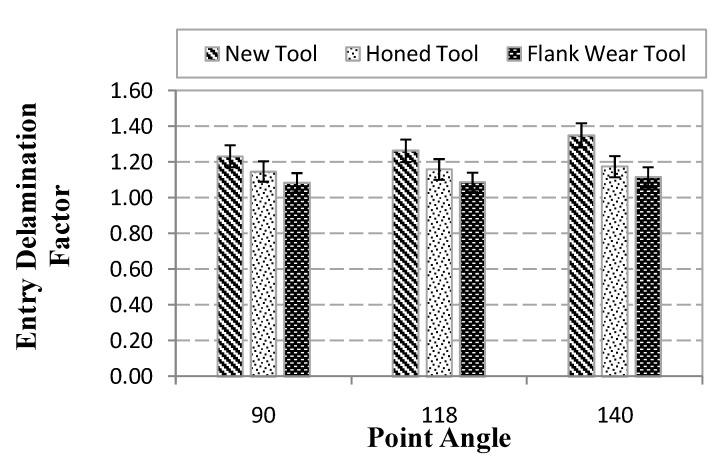
Maximum delamination at hole entry (measurement variations are indicated including error bars).

**Figure 8:**
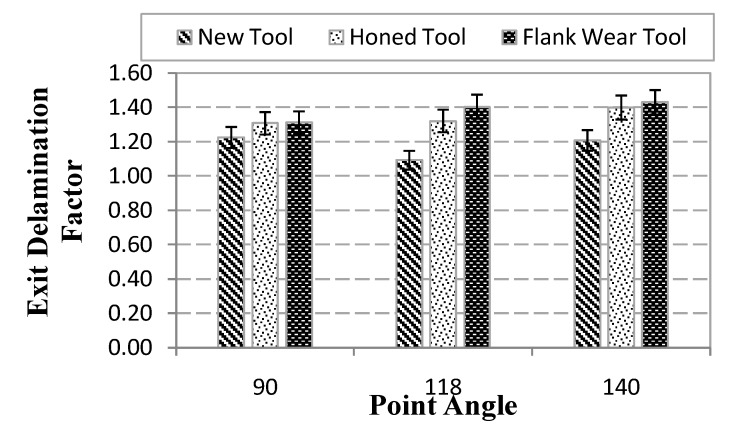
Maximum delamination at the hole exit (measurement variations are indicated including error bars).

The maximum delamination factor obtained after drilling tests at the hole entry and exit is presented in Figure 7 and Figure 8 respectively.

Delamination factor at the hole entry increased with point angle when drilling with a new tool. However, when a worn tool is used the influence of point angle is almost negligible. Concerning the influence of wear in the delamination factor at hole entry it is observed that the worn geometry produces less fraying of the fibers, and thus the extension of delamination is lower than in the case of fresh geometry. This behavior is consistent with the trends reported in [5] where similar effect was observed with angle point higher than 180°. The lowest delamination factor was obtained for drills exhibiting flank wear (in the range 1.14–1.18) while honed drills lead to slightly increased entrance delamination (around 1.3).

The delamination factor at the hole exit increased with wear, showing that the holes machined with flank wear the highest delaminated areas. This fact is related to the increment of the thrust force with wear progression.

The opposite effect of wear in the delamination factor considered at the hole exit and entrance can be explained as follows: when the cutting edges of drill bit make contact with the material, a peeling force through the slope of the drill results in separating the plies from each other. When the edge starts to wear, this force diminishes due to the decreasing sharpness. This fact elevates the thrust force but diminishes the peel force, leading to reduced peel up (related to hole entrance delamination) and elevated push out (corresponding to exit delamination) mechanisms.

Table 4 and Table 5 show the results of the ANOVA analysis corresponding to the entry and exit delamination factor. Drill geometry is the main factor with statistical and physical significance on delamination factor (*p*-value < 0.05 and Test F >> Fα = 5%). Feed rate and point angle might have a low contribution to delamination but not a negligible one (Test F > Fα = 5%). Finally, for both cases cutting speed is not a significant factor (*p*-value > 0.05 and Test F < Fα = 5%). Shyha* et al.* [12] and Davim* et al.* [18] came to the same conclusion with a similar ANOVA analysis for CFRP woven composites.

**Table 4:** ANOVA results for entry delamination factor.

Factors	SS	DF	MS	F	F *	P *
A: Geometry	0.485973	2	0.24299	60.58	3.12	**0.0000**
B: Point Angle	0.05018	2	0.02509	6.26	3.12	**0.0031**
C: Cutting Speed	0.020491	2	0.01025	2.55	3.12	0.0848
D: Feed rate	0.118565	2	0.05928	14.78	3.12	**0.0000**
Residual	0.28877	72	0.00401	–	–	–
Total	0.96398	80	–	–	–	–

SS: Sum of squares; DF: Degrees of freedom; MS: Mean square; F: F-test value; P *: Probability; * Significant at the 5% level.

**Table 5:** ANOVA results for exit delamination factor.

Factors	SS	DF	MS	F	F *	P *
A: Geometry	0.722067	2	0.361033	36.05	3.12	**0.0000**
B: Point Angle	0.108919	2	0.054459	5.44	3.12	**0.0063**
C: Cutting Speed	0.008956	2	0.004478	0.45	3.12	0.6412
D: Feed rate	0.138689	2	0.069344	6.92	3.12	**0.0018**
Residual	0.721059	72	0.010015	–	–	–
Total	1.69969	80	–	–	–	–

SS: Sum of squares; DF: Degrees of freedom; MS: Mean square; F: F-test value; P *: Probability; * Significant at the 5% level.

**Figure 9:**
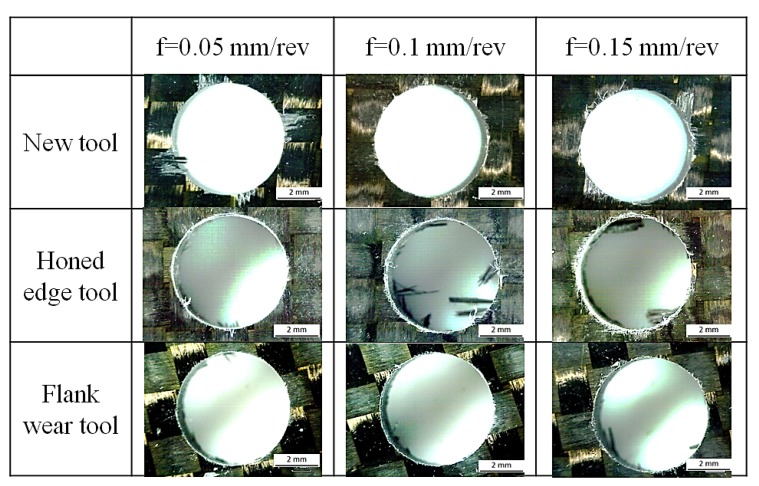
Hole entry quality variation with feed for different geometries with 180° point angle and *V* = 50 m/min.

It was possible to observe other defects related to the hole quality. Normally, fraying (Figure 9) was not observed at hole entrance for all geometries tested. Fraying was observed at the hole exit with both worn geometries. Flank wear produced less fraying than honed edge tool, and in some cases, it could be confused with chipping. It was observed that this defect increases when the point angle is higher, 118° and 140°. This defect showed a negligible dependence on feed.

**Figure 10:**
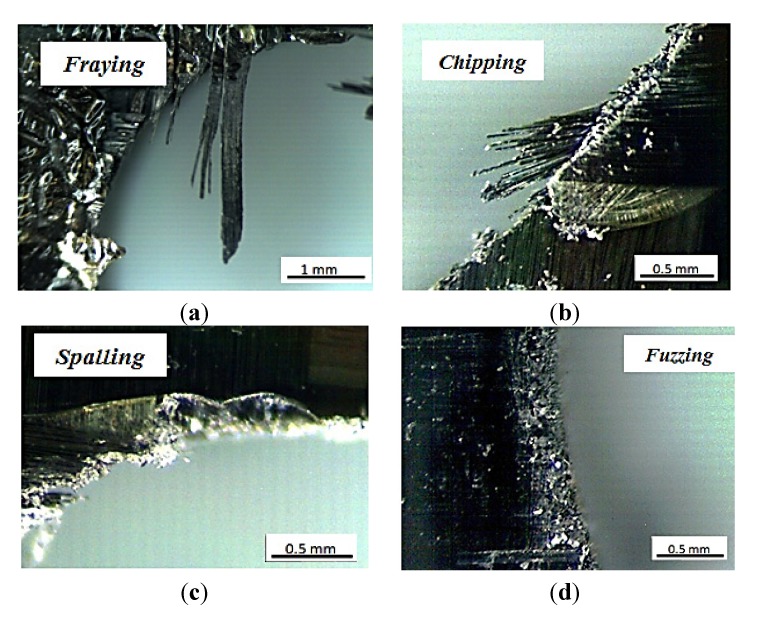
Different defects related with hole quality found after drilling. (**a**) fraying; (**b**) chipping; (**c**) spalling; and (**d**) fuzzing.

Concerning chipping (Figure 10), all geometries showed this defect both at the hole entry and exit. For worn tools, this defect was observed more frequently than for new tools and was enhanced for high feed. Increasing cutting speed has the same effect but less marked.

Another frequent surface defect was spalling, also observed with all geometries analyzed, but in different ways. For the honing tool, this defect was observed in a homogeneous area surrounding the hole entry, and located at some affected points at exit. Holes machined with flank wear and fresh tool showed this defect in concentrated zones at the hole entry and exit respectively, but it was more severe than that found with honing geometry. It was mainly observed with high feeds (0.15 mm/rev).

Finally, fuzzing (Figure 10) increased with point angle and feed and appeared mainly associated to machining with the honed edge. It was normally found together with spalling.

## 4. Conclusions

In this paper the influence of drill point angle and worn geometry in drilling of woven CFRP composite has been analyzed. The main contribution of the paper is the analysis of the crossed effect of the drill point angle and the geometrical changes due to wear progression. Moreover, the worn geometry was artificially generated ensuring the level and type of wear imposed and avoiding coupled effects of different wear patterns combined in the tool.

The following conclusions can be drawn:

Drill point angle influenced thrust force when it was combined with the effect of wear progression. However, fresh tools showed negligible influence of the drill point angle on thrust force. This fact is important for drill geometry selection since the evolution of wear could lead to inacceptable levels of thrust force.

Wear progression had a different effect on delamination at the entry and exit hole. While entry delamination diminished with wear progression, exit delamination was enhanced. The most favorable results concerning delamination were obtained with the lowest value of the drill point angle: delamination factor at entry and exit hole increased with the drill point angle. This result is important for the workpiece inspection after drilling, establishing critical zones. In addition, during the drill selection, the favorable effect of low drill point should be accounted for.

ANOVA analysis showed that the most influential parameters on thrust force and delamination were tool geometry (wear and point angle) and feed, while cutting speed has negligible influence. This result is important in order to define productive processes with high removal rates ensuring quality requirements of the workpiece.
